# Role of *Helicobacter pylori* infection in cancer‐associated fibroblast‐induced epithelial‐mesenchymal transition in vitro

**DOI:** 10.1111/hel.12538

**Published:** 2018-09-23

**Authors:** Gracjana Krzysiek‐Maczka, Aneta Targosz, Urszula Szczyrk, Malgorzata Strzałka, Zbigniew Sliwowski, Tomasz Brzozowski, Jarosław Czyz, Agata Ptak‐Belowska

**Affiliations:** ^1^ Department of Physiology The Faculty of Medicine Jagiellonian University Medical College Cracow Poland; ^2^ Department of Cell Biology The Faculty of Biochemistry, Biophysics and Biotechnology Jagiellonian University Cracow Poland

**Keywords:** cancer‐associated fibroblasts, E‐cadherin, epithelial‐mesenchymal transition, *Helicobacter pylori* infection

## Abstract

**Background:**

Major human gastrointestinal pathogen *Helicobacter pylori* (*H. pylori*) colonizes the gastric mucosa causing inflammation and severe complications including cancer, but the involvement of fibroblasts in the pathogenesis of these disorders in *H. pylori*‐infected stomach has been little studied. Normal stroma contains few fibroblasts, especially myofibroblasts. Their number rapidly increases in the reactive stroma surrounding inflammatory region and neoplastic tissue; however, the interaction between *H. pylori* and fibroblasts remains unknown. We determined the effect of coincubation of normal rat gastric fibroblasts with alive *H. pylori* (cagA+vacA+) and *H. pylori* (cagA−vacA−) strains on the differentiation of these fibroblasts into cells possessing characteristics of cancer‐associated fibroblasts (CAFs) able to induce epithelial‐mesenchymal transition (EMT) of normal rat gastric epithelial cells (RGM‐1).

**Materials and Methods:**

The panel of CAFs markers mRNA was analyzed in *H. pylori (cagA+vacA+)*‐infected fibroblasts by RT‐PCR. After insert coculture of differentiated fibroblasts with RGM‐1 cells from 24 up to 48, 72, and 96 hours, the mRNA expression for EMT‐associated genes was analyzed by RT‐PCR.

**Results:**

The mRNA expression for CAFs markers was significantly increased after 72 hours of infection with *H. pylori* (cagA+vacA+) but not *H. pylori* (cagA−vacA−) strain. Following coculture with CAFs, RGM‐1 cells showed significant decrease in E‐cadherin mRNA, and the parallel increase in the expression of Twist and Snail transcription factors mRNA was observed along with the overexpression of mRNAs for TGFβR, HGFR, FGFR, N‐cadherin, vimentin, α‐SMA, VEGF, and integrin‐β1.

**Conclusion:**

*Helicobacter pylori* (cagA+vacA+) strain induces differentiation of normal fibroblasts into CAFs, likely to initiate the EMT process in RGM‐1 epithelial cell line.

## 1 INTRODUCTION

Despite the incidence and mortality of gastric cancer (GC) have been decreasing, this disorder still remains one of the leading causes of cancer‐related death rate worldwide.^1^, ^2^, ^3^, ^4^ Despite the fact that the adjuvant chemotherapy and surgical resection are the only curative therapies nowadays, most patients are diagnosed with an advanced stage of disease due to lack of specific early symptoms. Furthermore, some patients lose the opportunity of curative resection resulting from the aggressive nature of GC. Although chemoradiotherapy and targeted therapy have confirmed an improvement in host response rates, the cancer recurrences and metastases are frequently observed.^2^, ^3^, ^4^, ^5^, ^6^ The bacteria *Helicobacter pylori* (*H. pylori)* is one of the major risk factors for GC development. Epidemiology of *H. pylori* indicates that this bug colonizes the human stomach of about 50% of the world's population. Although all *H. pylori*‐infected subjects develop gastritis, approximately 80% of these individuals remain asymptomatic.^7^, ^8^ Besides GC, *H. pylori* can also induce the gastric and duodenal ulcers and the mucosa‐associated lymphoid tissue (MALT) lymphomas affecting about 1%, 15%, and 0.1% of the population, respectively.^7^, ^8^ *H. pylori* colonizes mainly gastric epithelium but may also penetrate the mucus layer reaching pits of gastric glands.^9^ We have previously shown that fibroblasts may constitute a direct target for *H. pylori* 10 next to epithelial cells.^11^, ^12^ *H. pylori* colonization may directly and indirectly interact with fibroblasts, connective tissue, and other extracellular matrix components. Necchi et al^13^ have identified the presence of *H. pylori* not only in epithelial cells and intraepithelial intercellular spaces, but also in the underlying *lamina propria* and stromal tumor. This suggests that bacteria can alter the tight junctions and penetrate the deeper intercellular spaces down the underlying *lamina propria*. Moreover, the *H. pylori* infection increased the MMP‐7 expression, the number of myofibroblasts, and their proliferation and migration.^14^, ^15^ High MMP7 expression facilitated cancer invasion and angiogenesis by degrading extracellular matrix macromolecules and connective tissues in vivo. Recently, the direct interaction between this bacterial pathogen and fibroblasts has been proposed^16^ suggesting that *H. pylori* can interact with several components of connective tissue components including fibroblasts. The most virulent *H. pylori* strains have been shown to harbor the cag pathogenicity island encoding the type IV secretion system,^3^, ^17^ allowing the delivery of bacterial cytotoxins into gastric epithelial cells, inducing phenotypic alterations reminiscent of an epithelial to mesenchymal transition (EMT).^3^, ^17^, ^18^, ^19^

The EMT is a biological process in which polarized epithelial cells lose the adherence and tight cell‐cell junction, enhance their migratory capacity, and become resistant to apoptosis.^20^ Moreover, the EMT increased the production of components of extracellular matrix (ECM) and gained the invasive properties to become mesenchymal cells known to play an essential role in cancer progression and metastasis.^21^, ^22^, ^23^, ^24^ EMT allows the tumor cells to acquire invasive properties and to develop metastatic growth characteristics.^21^, ^23^ These events are facilitated by the reduction in cell‐cell adhesion molecule E‐cadherin, the upregulation of more plastic mesenchymal proteins such as vimentin, N‐cadherin, and α‐SMA and deregulation of the Wnt pathway.^23^, ^24^ Many EMT‐inducing transcription factors (EMT‐TFs) such as Twist1, Snail1, Snail2, Zeb1, and Zeb2 can repress E‐cadherin both directly or indirectly.^23^, ^24^, ^25^, ^26^

Interestingly, the eradication of *H. pylori* leads to the reduction in the expression of TGF‐β1, Twist, Snail, Slug, and vimentin mRNAs, while enhancing the expression of E‐cadherin. This suggests that *H. pylori* infection may trigger the TGF‐β1‐induced EMT pathway and that *H. pylori* eradication may inhibit the GC progression by attenuation of this pathway.^27^, ^28^

The activated myofibroblasts accompanying tumors known as cancer‐associated fibroblasts (CAFs) belong to the principal constituents of the tumor stroma, playing important role in the tumor microenvironment.^29^ The CAFs were shown to mediate cancer‐related inflammation by expressing proinflammatory and tumor‐promoting factors and promotion of the cancer cell invasion and ECM remodeling.^30^, ^31^ Moreover, under the control of a variety of stroma‐modulating factors, the cancer cells themselves generate a permissive microenvironment favoring further tumor development and invasion.^32^, ^33^, ^34^

The proinflammatory factors released by CAFs, such as IL‐6, COX‐2 and CXCL1, FSP1, CXCL9, CXCL10 (IP‐10), and CXCL12 (SDF‐1 stromal cell‐derived factor 1), were implicated in the mechanism of tumor growth and neoplastic cell invasion.^35^, ^36^, ^37^, ^38^, ^39^ The CAFs secrete proangiogenic factors, such as IL‐8, SDF‐1, vascular endothelial factor (VEGF), and fibroblast growth factor (FGF), into an environment of other stromal cells including endothelial cells to promote tumor angiogenesis.^30^, ^35^, ^38^, ^39^ CAFs may enhance invasion of the cancer cells through expression of TGF β, potent EMT inducer, and HGF, which has been shown to promote breast tumorigenesis.^39^, ^40^ Since fibroblasts may alter the mRNA expression of structural and cell cycle‐associated genes in the presence of *H. pylori*,^9^, ^41^ we have attempted to determine whether *H. pylori* can interact with fibroblasts by changing them not only into myofibroblasts, but also into CAFs, further being capable of inducing EMT program in normal RGM‐1 epithelial cell line.

## 2 MATERIALS AND METHODS

### 2.1 Bacterial *H. pylori* strains and their characterization

The *H. pylori* strain expressing CagA and VacA cytotoxins (43504 *H. pylori cagA+vacA+ (s1/m1)*) was purchased from American Type Culture Collection. The *H. pylori* strain negative for CagA and VacA (*H. pylori cagA−vacA− (s2/m2)* 42 originated from our bacterial bank of isolations from gastric biopsy specimens of the patients with gastric ulcer who underwent upper endoscopy. The genomic DNA was isolated from *H. pylori* strains using Genomic mini (A&A Biotechnology, Gdynia, Poland). For each single PCR, 20 μg of DNA was used. Specific primers for the detection of *cagA* and *vacA* were used (Sigma‐Aldrich, Poznan, Poland). The sequences of primers are listed in Tables A1 and A2 (see Appendix I). Stock cultures were maintained at −70°C in Brucella broth (Becton Dickinson, Sparks, MD, USA) supplemented with 10% fetal bovine serum and 10% glycerol.

The cultures of bacteria were grown on Columbia agar with 5% fresh horse blood (BioMerieux, Warsaw, Poland). The plates were incubated under microaerophilic conditions at 37°C for 3‐5 days.

### 2.2 Technique of rat gastric fibroblast isolation and the infection of isolated fibroblasts with *H. pylori*

Gastric samples were harvested from 8‐week‐old Sprague‐Dawley rats and extensively washed with sterile PBS to remove contaminating debris. Primary fibroblast culture was established by mincing gastric biopsy into 1‐ to 2‐mm^3^ pieces with scissors and placing it in tissue culture flasks under sterile conditions. Growth medium DMEM containing 10% FBS and antibiotics were added and gently mixed with minced tissue. The flasks were maintained in a humidified atmosphere of 5% CO_2_ at 37°C, and the medium was changed every 2 days. When the cells grew up to 70% of confluence, they were passaged using standard trypsinization techniques to establish a secondary cell culture as reported before.^9^, ^43^, ^44^

Before the coincubation with fibroblasts, *H. pylori* strains were first suspended in sterile PBS and immediately transferred to the dishes containing fibroblasts. The 70% confluent fibroblasts were infected with 1 × 10^9^ of live *H. pylori* per dish and incubated in humidified atmosphere for 72 hours.

### 2.3 Determination of mRNA expression by RT‐PCR

After incubation period, total cellular RNA was isolated according to Chomczynski and Sacchi method^45^ using Trizol reagent (Invitrogen, Carlsbad, CA, USA). First‐strand cDNA was synthesized from total cellular RNA (2 μg) using Reverse Transcription System (Promega, Mannheim, Germany). The PCR was carried out in an automatic DNA thermal cycler, using 1 μg cDNA and Promega PCR reagents. Expression of transcripts in the rat gastric fibroblasts and RGM‐1 cell line (Ricken Bank, Tsukuba, Ibaraki, Japan) was determined by RT‐PCR using specific primers (Sigma‐Aldrich). Amplification of control rat 18s RNA was performed on the same samples to verify the RNA integrity. PCR products were separated by electrophoresis in 2% agarose gel containing 0.5 μg/mL ethidium bromide and then visualized under UV light. Location of predicted PCR product was confirmed by using O'Gene Ruler 50‐bp DNA ladder (Fermentas, Life Sciences, Waltham, MA, United States) as standard marker.

### 2.4 Markers of fibroblast to CAF transdifferentiation

The influence of *H. pylori* (cagA−vacA−) strain selected from our bank of nine different samples collected from *H. pylori*‐infected patients on fibroblasts was determined by the analysis of expression of β‐actin, α‐SMA, collagen I, HIF‐1α, and HSP‐70 mRNA. The effects of *H. pylori* (cagA−vacA−) strain were compared with those exerted by *H. pylori* (cagA+vacA+) strain. After 72 hours of coculture of *H. pylori* (cagA+vacA+) with fibroblasts, the cells were harvested and total cellular RNA was isolated as mentioned above. Expression of 18s, α‐SMA, collagen I, collagen III, tenascin C (TNC), FAP, FSP, IL‐6, IL‐8, SDF‐1, HGF, TGFβ, and COX‐2 transcripts in the rat gastric fibroblasts were determined by RT‐PCR using specific primers (Sigma‐Aldrich; Table A1, Appendix I).

### 2.5 Coculture of epithelial cells (RGM‐1) with fibroblasts after 72 hours of their incubation with *H. pylori (*cagA+vacA+)

**Figure 1:**
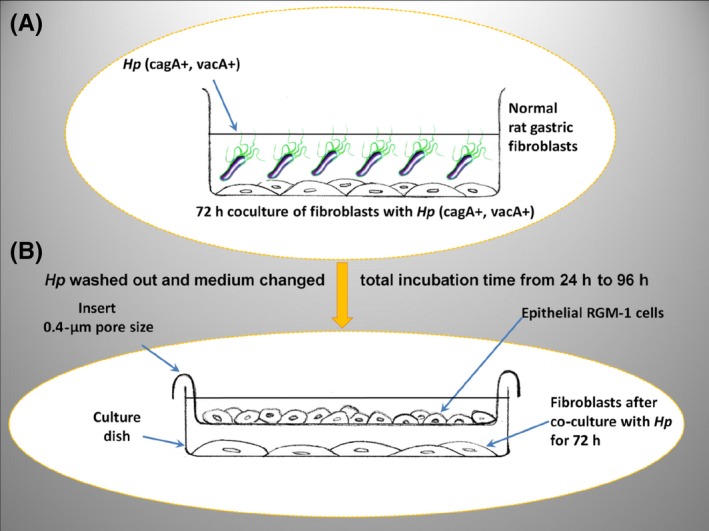
The insert coculture of fibroblasts with epithelial RGM‐1 cells. After 72 h of fibroblast coculture with *Helicobacter pylori* (cagA+vacA+) (A), the *H. pylori* was washed out and medium changed into RPMI with 10% FBS and antibiotics. The 0.4‐μm pore size cell culture inserts (Becton Dickinson) were placed on the layer of fibroblasts and medium. Trypsinized RGM‐1 cells were then seeded on the inserts surface, the medium was filled up to 5 mL and the epithelial cells were subsequently coincubated with fibroblasts in humidified atmosphere for 24, 48, 72, and 96 h (B)

After 72 hours of fibroblast coculture with *H. pylori (*cagA+vacA+), the *H. pylori* was washed out from fibroblasts and the medium was changed into DMEM with 10% FBS and antibiotics. The culture dish was maintained in a humidified atmosphere of 5% CO_2_ at 37°C for 4 hours, and then, the incubatory fluid was again replaced with fresh portion f the medium. On the layer of fibroblasts and medium, 0.4‐μm pore size cell culture inserts (Becton Dickinson) were placed (Figure 1A,B).

Trypsinized RGM‐1 cells were then seeded on the inserts surface, the medium was filled up to the volume of 5 mL, and the cells were coincubated in humidified atmosphere for 24, 48, 72, and 96 hours (Figure 1B). After incubation period, the total cellular RNA from RGM‐1 cells was isolated as described above.^45^

### 2.6 Determination of markers of epithelial to mesenchymal transition in RGM‐1 cells

The expression of 18s, α‐SMA, N‐cadherin, vimentin, E‐cadherin, β1‐integrin, COX2, VEGF, TGFBR, HGFR, FGFR, Snail, Twist, Ki67, Bax, Bcl‐2 transcripts in the rat gastric fibroblasts were determined by RT‐PCR, using specific primers (Sigma‐Aldrich; Table A1, Appendix I).

### 2.7 Contrast‐phase microscopy

For microscopic examination, the trypsinized fibroblasts were seeded on cover glasses and cocultured with and without presence of *H. pylori* (cagA+vacA+) for 72 hours in antibiotic‐free DMEM. Then, the medium with *H. pylori* was removed and the fresh DMEM with 10% FBS and antibiotics was added. After 3 hours, medium has been changed again for the fresh one and left for 96 hours. After the end of 96 hours of incubation, the medium from fibroblasts cocultured with or without *H. pylori* was collected. Trypsinized RGM‐1 cells were seeded on cover glasses and then cultured in the presence of the collected supernatant from *H. pylori*‐infected fibroblasts for 24, 48, 72, and 96 hours. As the control, RGM‐1 cells cultured in the supernatant from 96 hours culture of *H. pylori*‐noninfected fibroblasts in DMEM with 10% FBS and antibiotics were used. Both fibroblasts and RGM‐1 cells were fixed in 3.7% formaldehyde for 20 minutes at room temperature. The image acquisition was performed with a Leica DMI6000B microscope (Leica Microsystems, Wetzlar, Germany).

### 2.8 Statistical analyses

Statistical analysis of the data was performed with the use of Excel Software. Results are expressed as means ± SEM from six samples per each group. Statistical significance of difference was determined using analysis of variance (one‐way ANOVA) test (Statistica Software, StatSoft, Cracow, Poland). Further statistical analysis for post hoc comparisons was carried out with Newman‐Keuls test. Differences were considered statistically at *P* < 0.05.

## 3 RESULTS

### 3.1 Expression of markers of activation and differentiation of fibroblasts

**Figure 2:**
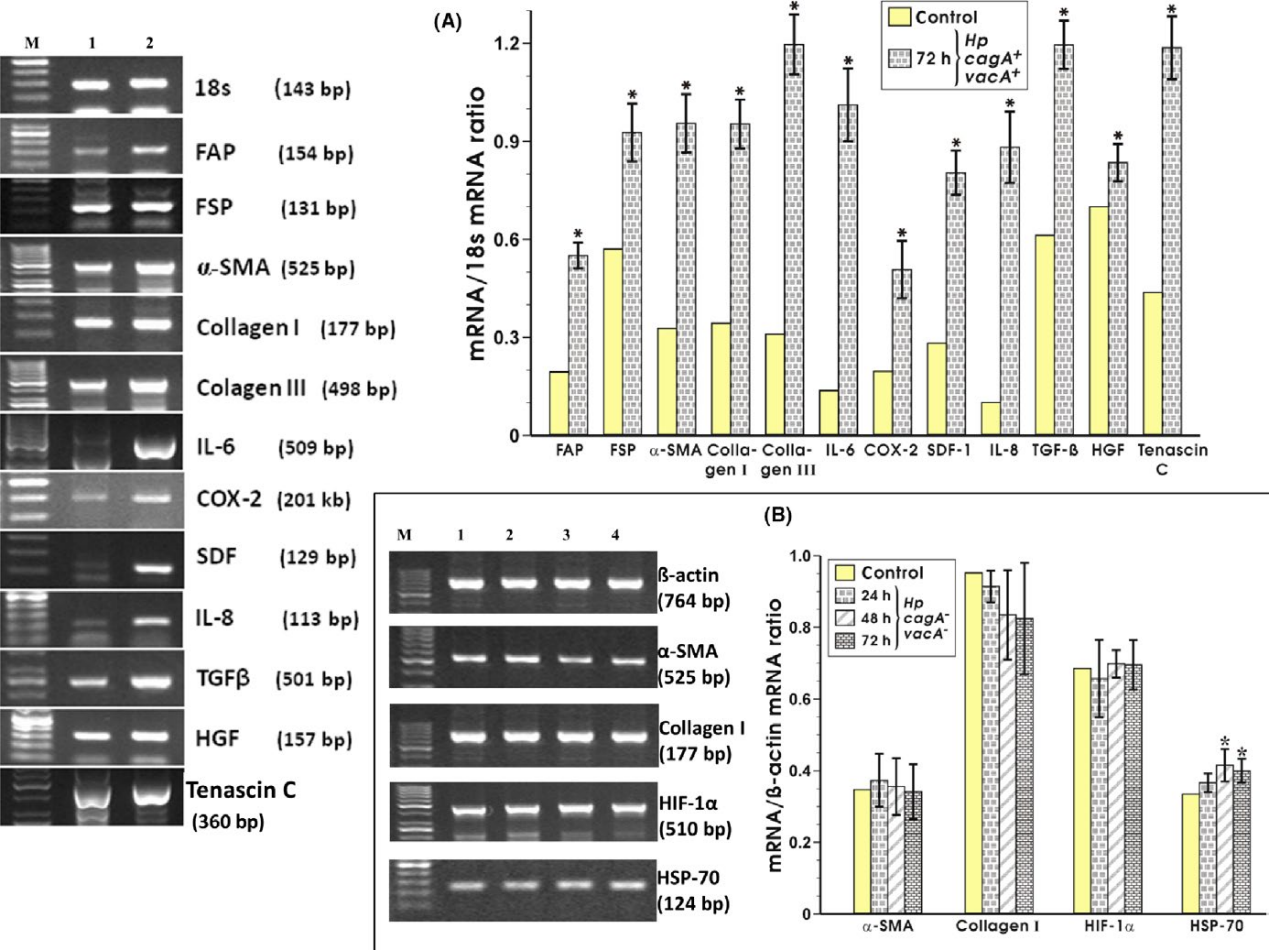
RT‐PCR analysis of expression of 18s RNA and a panel of CAF markers mRNA expression in rat gastric fibroblasts after 72 h of coincubation with *Helicobacter pylori* strain cagA+vacA+ and the semiquantitative ratio of selected genes over 18s after 72 h of coincubation with this bacteria (panel A). RT‐PCR determination of β‐actin and markers of the fibroblasts differentiation after 24, 48, and 72 h of rat gastric fibroblasts coincubated with *H. pylori* (cagA−vacA−) strain and the ratio of selected genes over β‐actin after 24, 48, and 72 h of coincubation with this strain (panel B). Results are mean ± SEM of six determinations. Asterisk indicates a significant change (*P* < 0.05) as compared to the control value

The exposure of fibroblasts to *H. pylori* (cagA+vacA+) for 72 hours resulted in a significant rise in FAP and FSP mRNA expression comparing with those obtained in control fibroblasts cultured without *H. pylori* (Figure 2A). Differentiation of fibroblasts in the presence of *H. pylori* was confirmed by an increase in expression of α‐SMA, collagen I, and collagen III. *H. pylori*‐infected fibroblasts showed a significant increase as compared with control fibroblasts without *H. pylori* infection (Figure 2A). As shown in Figure 2B, the coculture of gastric fibroblasts with *H. pylori* (cagA−vacA−) strain failed to alter their differentiation as reflected by the lack of significant changes in mRNA expression of α‐SMA and collagen I at 24, 48, and 72 hours of incubation. Also, HIF‐1α mRNA expression was not significantly changed. However, the slight but significant increase in expression of HSP‐70 mRNA has been observed at 48 hours of fibroblasts incubation with *H. pylori* (cagA−vacA−). Therefore, for further *H. pylori* influence examination, only *H. pylori* (cagA+vacA+) was selected.

### 3.2 Proinflammatory factors and angiogenic potential markers expressed by rat gastric fibroblasts

One of the CAFs potential characteristics is their ability to secrete proinflammatory cytokines, known to facilitate the recruitment of granulocytes and lymphocytes and to promote tumor growth and metastasis. Therefore, we determined the expression of IL‐6, IL‐8, COX‐2, and SDF‐1 mRNA in fibroblasts infected with *H. pylori* (cagA+vacA+). After 72 hours of coincubation with bacteria, an induction of IL‐6 and SDF‐1 mRNA expression as compared with control fibroblasts was observed (Figure 2A). We have also noticed a highly significant increase in mRNA expression of COX‐2 and IL‐8 in cells infected with *H. pylori* (cagA+vacA+) strain (Figure 2A).

### 3.3 EMT inducers, ECM components, and proinvasive signals

The mRNA expression for TGFβ and scattering factor HGF was significantly increased at 72 hours of coincubation with *H. pylori* (cagA+vacA+) over those observed in control fibroblasts (Figure 2A). Since CAFs produce a variety of ECM proteins, which are the structural components involved in making up of connective tissue and under pathological conditions contribute to the dense fibrous nature of solid tumors, we have determined the expression of mRNAs for these proteins in fibroblasts cocultured with or without *H. pylori*. At 72 hours of coincubation with *H. pylori* positive for cagA and vacA, a significant increase in TNC mRNA expression besides the rise in the expression of collagen I and III has been observed (Figure 2A).

### 3.4 Morphology of fibroblasts

**Figure 3:**
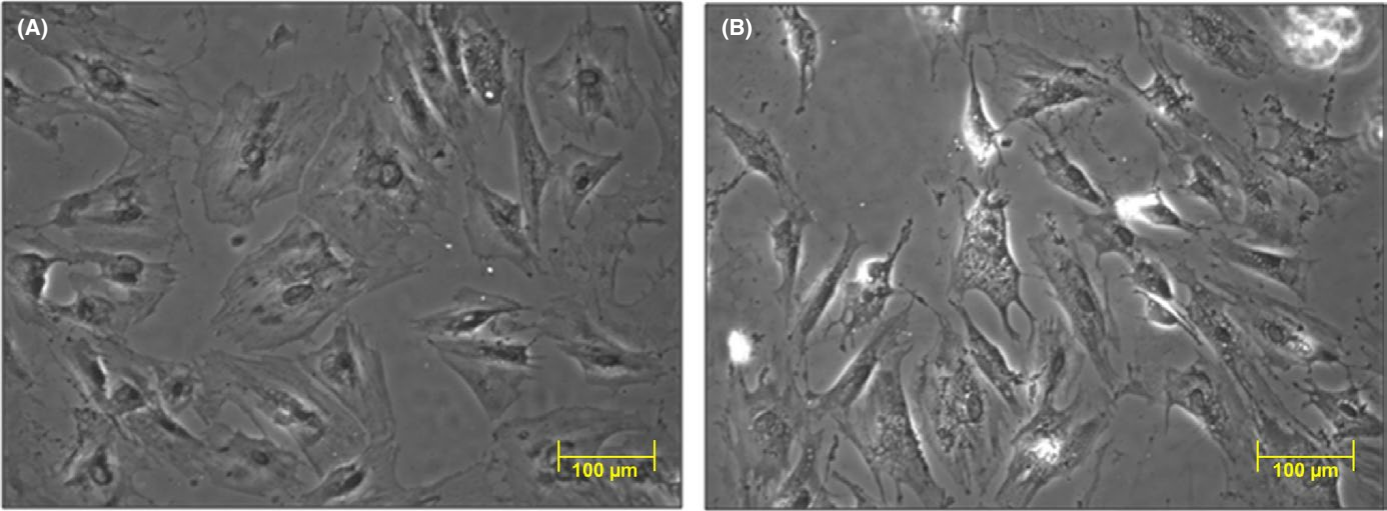
Morphology of isolated normal gastric fibroblasts (A) and of fibroblasts after 72 h coculture with *Helicobacter pylori* (cagA+vacA+) (B). Contrast‐phase microscopy view showing the difference in morphology of both cultures of fibroblasts as indicated by elongated shape and abundant protrusions (arrows) (B vs A)

Comparing to control fibroblasts, those cultured in the presence of *H. pylori* (cagA+vacA+) revealed prominent morphological changes characterized by elongated shape and the presence of abundant protrusions indicating their activation and phenotypical alterations (Figure 3A,B).

### 3.5 Process of epithelial to mesenchymal transition in epithelial RGM‐1 cell line cocultured with *H. pylori* (cagA+vacA+)‐treated fibroblasts compared with control fibroblasts noninfected with *H. pylori*

#### 3.5.1 Expression of mesenchymal markers

**Figure 4:**
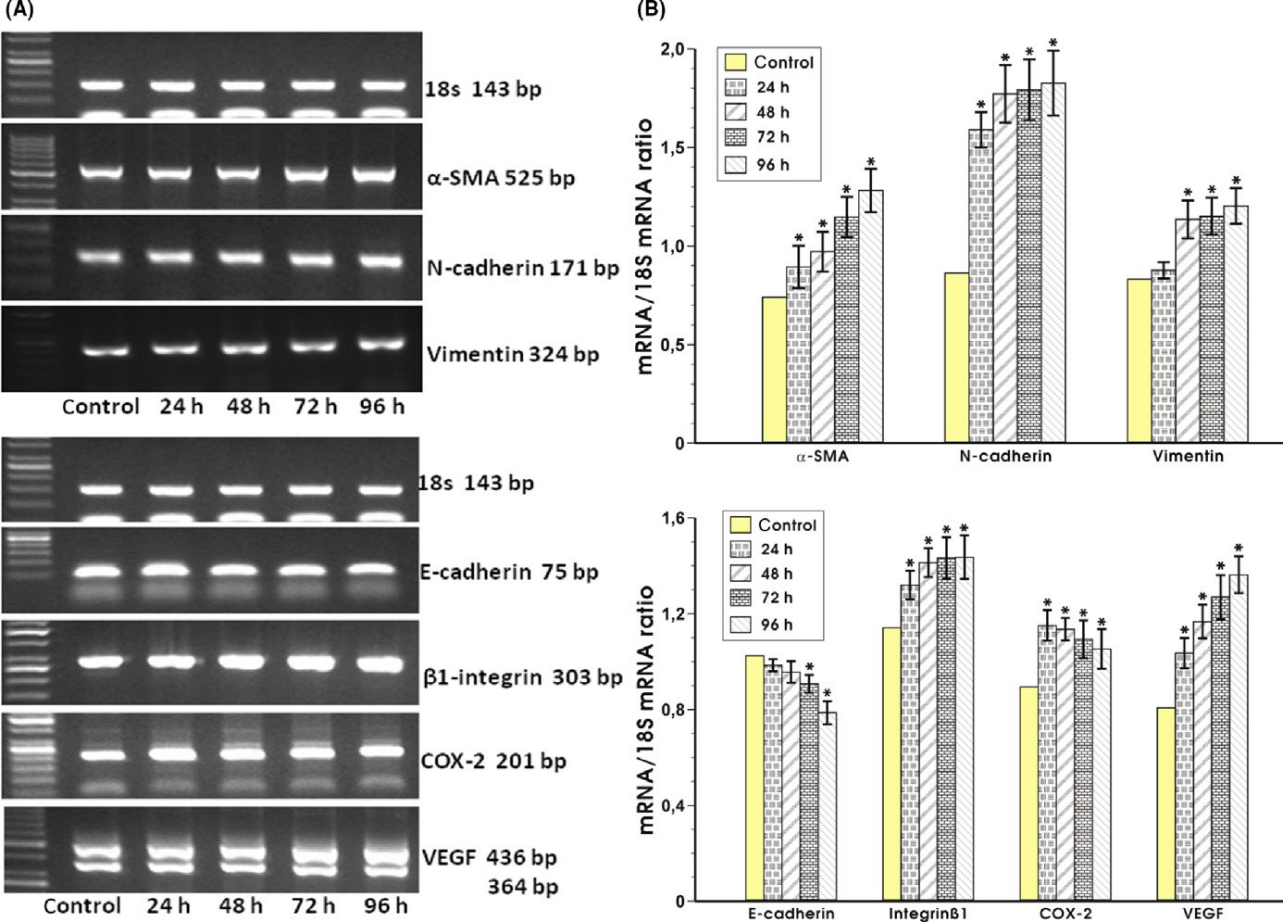
The RT‐PCR analysis of mRNA expression for mesenchymal markers (α‐SMA, N‐cadherin, vimentin) and promigrative and proangiogenic factors (E‐cadherin, β1‐integrin, COX‐2, VEGF) standardized to 18s RNA in rat epithelial RGM‐1 cells after 24, 48, 72, and 96 h of coculture with fibroblasts which were initially infected with *Helicobacter pylori* strain (cagA+vacA+) for 72 h (panel A) and the semiquantitative ratio of selected mRNAs over 18s mRNA after coculture of these epithelial cells with *H. pylori*‐infected or noninfected (control) fibroblasts (panel B). Results are mean ± SEM of six determinations. Asterisk indicates a significant (*P* < 0.05) change as compared to the control value

The expression of α‐SMA was significantly increased at 24 hours, with further progression in time up to 96 hours in fibroblasts cocultured with *H. pylori* (Figure 4A,B). The N‐cadherin mRNA expression showed a significant increase already after 24 hours of coculture, the effect which persisted up to 96 hours of incubation (Figure 4A,B). The expression of mRNA for vimentin was significantly increased in epithelial cells after 48 hours of coculture with *H. pylori*‐treated fibroblasts and remained elevated up to 96 hours. All analyses were performed in comparison with RGM‐1 cells cocultured with control fibroblasts noninfected with *H. pylori*.

#### 3.5.2 Expression of promigrative and proangiogenic factors

The statistically significant transcriptional suppression of the adhesion molecule E‐cadherin was noticed between 48 and 72 hours of RGM‐1 cells cocultured with *H. pylori*‐infected fibroblasts compared to control coculture. This suppression increased at 96 hours of coculture suggesting a progressive downregulation of E‐cadherin over the time of incubation (Figure 4A,B). In order to estimate possible increase in migrative properties of RGM‐1 cells, we have measured the β1‐integrin expression of mRNA. The coincubation RGM1 cells with *H. pylori*‐infected fibroblasts resulted in a significant increase in β1‐integrin mRNA expression at 24 hours which was sustained over the time of observation up to 96 hours as compared with control coculture (Figure 4A,B). In addition, the mRNA expression for COX‐2, the factor considered as an accelerator of invasion, metastasis, and angiogenesis, was significantly increased at 24 hours following coculture with *H. pylori*‐infected fibroblasts compared with control (Figure 4A,B). The increase in COX‐2 mRNA expression was sustained up to 96 hours of incubation (Figure 4A,B).

The angiogenic potential was evaluated by determination of VEGF mRNA expression. Consistently, the VEGF mRNA showed a significant increase at 24 hours with further increase with time as compared with control coculture without *H. pylori* infection (Figure 4A,B).

#### 3.5.3 Expression of EMT triggering signals

**Figure 5:**
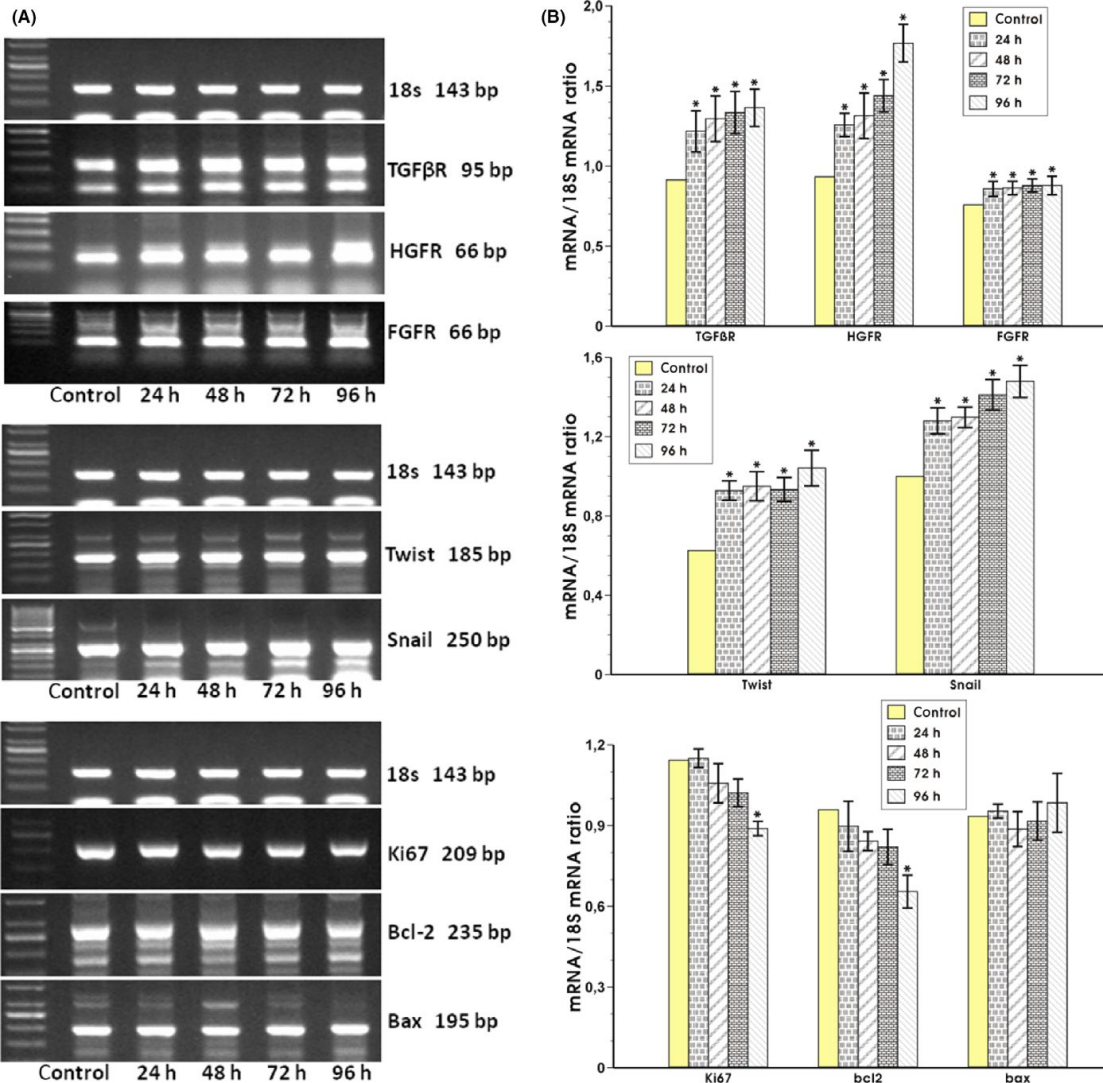
The RT‐PCR analysis of mRNA expression for receptors for EMT triggering factors (TGFβR, HGFR, FGFR), transcription factors (Twist, Snail), and proliferation and apoptosis regulators (Ki67, Bcl‐2, Bax) in rat epithelial RGM‐1 cells standardized to 18s RNA in rat epithelial RGM‐1 cells after 24, 48, 72, and 96 h of coculture with fibroblasts which were initially infected with *Helicobacter pylori* strain (cagA+vacA+) for 72 h (panel A) and the semiquantitative ratio of selected mRNAs over 18s mRNA after coculture of these epithelial cells with *H. pylori*‐infected or noninfected (control) fibroblasts (panel B). Results are mean ± SEM of six determinations. Asterisk indicates a significant (*P* < 0.05) change as compared to the control value

The expression of the mRNAs for growth factors receptors for TGFβ, HGF, and FGF known as the EMT triggering signals was determined. The TGFβR and FGFR mRNA expressions were strongly upregulated from 24 hours up to 96 hours of incubation with *H. pylori*‐infected fibroblasts compared with control coculture (Figure 5A,B). The significant increase in mRNA expression for HGFR was also observed after 24 hours with further increase within time up to 96 hours of incubation compared with control coculture (Figure 5A,B).

#### 3.5.4 Expression of transcription factors specific for induction of EMT process, proliferation, and apoptosis

The expression of mRNA for transcription factors Twist and Snail was significantly increased at 24 hours of epithelial cells coculture with *H. pylori*‐infected fibroblasts. Twist mRNA expression remained elevated up to 96 hours (Figure 5A,B) and the Snail mRNA expression increased over the time of observation up to 96 hours as compared with control coculture (Figure 5A,B). The mRNA expression for proliferation indicator Ki67 significantly decreased at 72 and 96 hours of incubation with *H. pylori*‐infected fibroblasts in comparison with control coculture (Figure 5A,B). The mRNA for proapoptotic Bax was not significantly altered at any of the time periods while expression of mRNA for antiapoptotic Bcl‐2 showed a significant decrease at 96 hours as compared to control coculture (Figure 5A,B).

### 3.6 Morphology of RGM‐1 cells

**Figure 6:**
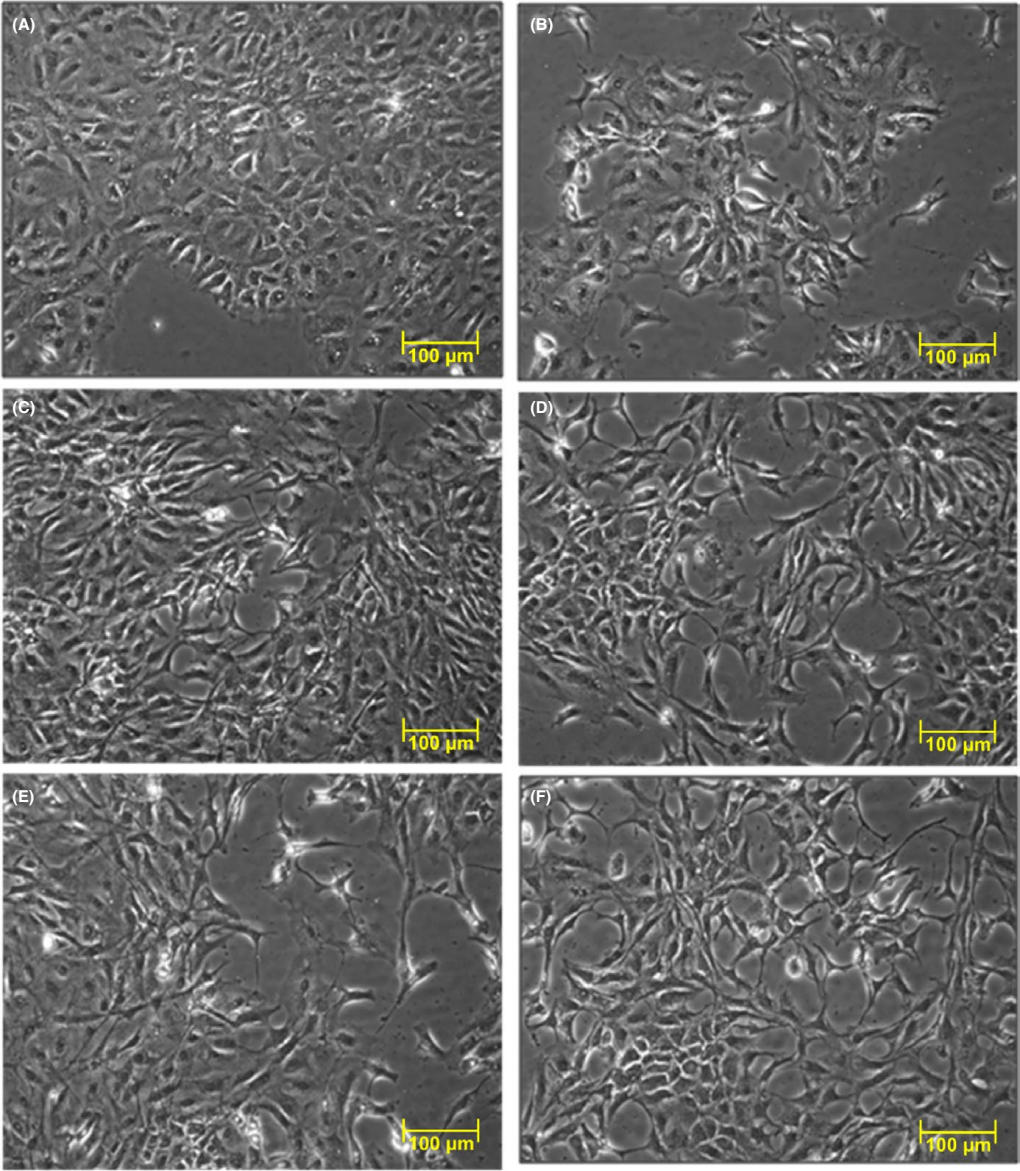
Contrast‐phase microscopy view showing control epithelial RGM‐1 cells cultured in DMEM + 10% FBS + antibiotics (A), control epithelial RGM‐1 cells cultured in supernatant from 96 h culture of normal, noninfected fibroblasts (B) and RGM‐1 cells cultured for 24, 48, 72, and 96 h respectively, in supernatant from 96 h culture of fibroblasts previously infected with *Helicobacter pylori* for 72 h (DMEM + 10% FBS + antibiotics) (C‐F). The changes in morphology of epithelial cells and disorders in continuity of monolayers were observed already after 24 h of the culture in the supernatant from fibroblasts infected with *H. pylori* (C) and have been particularly apparent at 96 h of culture in this supernatant (F) comparing to control conditions (A, B)

Depending on culture conditions, the RGM‐1 cells show prominent morphological changes (Figure 6A‐F). RGM‐1 cells cultured in the presence of the supernatant from 96 hours culture of fibroblasts infected for 72 hours with *H. pylori* (cagA+vacA+) became elongated with prominent protrusions. This evidently suggests their activation and phenotypical changes. These alterations occurred already after 24 hours of incubation with the supernatant from *H. pylori*‐infected fibroblasts (Figure 6C‐F). Differences in RGM‐1 cells were particularly apparent at 96 hours (Figure 6F) suggesting progression of these changes with time. We have also observed distortion of the monolayer continuity starting at 24 hours of the RGM1 culture with the *H. pylori*‐infected fibroblasts supernatant (Figure 6C) comparing with control conditions (Figure 6A,B), with the most prominent effect being observed at 96 hours of incubation (Figure 6F).

### 3.7 Identification of *H. pylori* (cagA−vacA−) strain used for comparison with *H. pylori* (cagA+vacA+) strain

**Figure A1:**
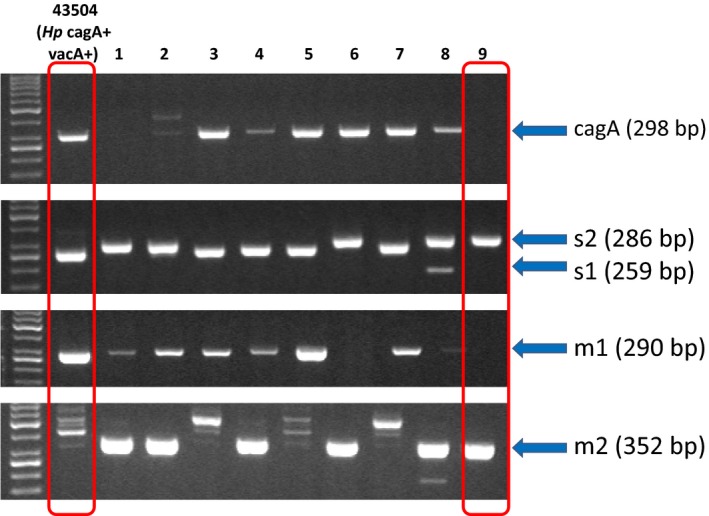
Detection of *Helicobacter pylori* cagA and vacA (m1, m2, s1, and s2) DNA regions in strains isolated from nine different samples collected from *H. pylori*‐infected patients as compared with strain *H. pylori* ATCC 43504 cagA+vacA+ (m1, s1 positive) used as the control for cagA and s1, m1 vacA regions expressing a highest vacA activity (left red frame). The sample nr 9 indicates type s2 and m2 (m2s2) strain which was chosen as being cagA negative and bearing the lowest vacA cytotoxin expression (*H. pylori* cagA−vacA−) (right red frame)

Figure A1 (Appendix I) shows the detection of *H. pylori* cagA and vacA (m1, m2, s1, and s2) DNA regions in strains isolated from nine different samples collected from *H. pylori*‐infected patients. For this purpose, we have utilized strain *H. pylori* ATCC 43504 cagA+vacA+ (m1, s1 positive) for cagA and s1, m1 vacA regions expressing a highest vacA activity (left red frame). As shown in Figure A1, the sample number 9 indicates type s2 and m2 (m2s2) strain which was chosen as being cagA negative and bearing the lowest vacA cytotoxin expression (*H. pylori* cagA−vacA−; right red frame).

## 4 DISCUSSION

Previous studies have established a key role of *H. pylori* in the pathogenesis of a variety of gastric diseases including gastritis, peptic ulcers, B lymphoma, and gastric cancer.^1^, ^2^, ^3^, ^4^, ^7^, ^8^ The impaired balance between *H. pylori*‐producing aggressive bacterial endotoxins and host defensive mechanisms is critical in the different clinical outcomes observed in patients infected with this bug. Recently, we have reported that gastric fibroblasts can represent cellular target of *H. pylori*.^10^ The increased fibroblasts to myofibroblasts differentiation with increased collagen production, the alteration in proliferation, and apoptosis were implicated in the mechanism of interaction between fibroblasts and *H. pylori* (cagA+vacA+).^10^ This suggests that stroma cells may constitute a novel target of *H. pylori* activity.^10^

It has been demonstrated that further transformation of myofibroblasts and fibroblasts into CAFs as well as the interactions between the neoplastic and non‐neoplastic cells with the ECM leads to the extensive desmoplastic reaction. CAFs play an important role in tumor progression.^39^, ^46^, ^47^ The coinjection of CAFs with tumor cells enhanced tumor formation which was uncommon after the coinjection of these tumor cells with normal fibroblasts.^46^, ^47^ Therefore, we have addressed the question whether isolated fibroblasts can acquire the characteristic of CAFs when coincubated with cytotoxic cagA+vacA+ strain of *H. pylori*.

The formation of CAFs involves several important features: (a) the expression of fibroblast markers vimentin, FSP1 and FAP; (b) the expression of activation marker α‐SMA; (c) the expression of aggressive/invasive markers including TNC; (d) an increase in cytokine and growth factor expression including IL‐8, IL‐6, SDF‐1, VEGF, TGF‐β, HGF, and FGF‐2; and (e) the reprogramming of ECM proteins and matrix remodeling proteins.^4^, ^35^, ^48^, ^49^, ^50^, ^51^, ^52^, ^53^ Interestingly, we have observed that the mRNA expression of α‐SMA, collagen I, or HIF‐1α was not altered by *H. pylori (*cagA−vacA−) in contrast to the moderate increase in HSP‐70 mRNA expression possibly due to the “cytoprotective” properties of bacterial wall product LPS.^54^, ^55^ In the case of *H. pylori* (cagA+vacA+), we have observed a strong upregulation of mRNA for α‐SMA along with overexpression of FAP mRNA in fibroblasts. The membrane gelatinase FAP belonging to the serine protease family has been implicated in extracellular matrix remodeling^52^, ^53^ as well as in the regulation of fibroblast growth and EMT interactions during development, tissue repair, and epithelial carcinogenesis.^52^, ^53^, ^56^ The FAP‐positive CAFs enhanced the survival, proliferation, and migration of gastric cancer cell (GC) line in vitro, in addition of inducing a drug resistance of the GC cells in the gastric cancer patients.^53^ Since FAP is upregulated in stroma fibroblasts of over 90% of malignant epithelial tumors, this factor may serve as a potential biomarker and therapeutic target of stromal tumors.^56^ Furthermore, the FAP‐expressing CAFs increased expression of proinflammatory genes, thus promoting tumor immune evasion in a mouse model of pancreatic cancer.^57^

When fibroblasts were coincubated with *H. pylori* (cagA+vacA+), the increased expression of FSP mRNA has been observed in our present study. FSP is considered as a marker of fibroblasts in different organs undergoing tissue remodeling and helps to identify fibroblasts derived from EMT in several organs including the liver.^58^ *H. pylori* can stimulate TLRs leading to activation of innate immunity against bacterial inflammation, thus creating an inflammatory microenvironment, a prerequisite for tumor development through activation of NF‐κB and STAT3 pathways in human GC.^59^, ^60^ Herein, we have shown that possible downstream targets of NFκB and STAT3 pathways including TGFβ, HGF, IL‐6, IL‐8, TNC, collagens I and III were upregulated in fibroblasts infected by *H. pylori* (cagA+vacA+). We have also observed a significant induction of COX‐2 mRNA expression, which is closely associated with the formation and development of majority of gastric cancers.^61^, ^62^

The expression of IL‐6 and IL‐8 mRNAs was implicated in the early stage of tumorigenesis.^35^, ^63^ The media collected from GC lines have induced IL‐6 secretion in fibroblasts considered as the paracrine stimulant of proliferation of tumor gastric cells through the activation of STAT3 signaling.^63^ Besides, the upregulation of IL‐6 after the coincubation of fibroblasts with *H. pylori* (cagA+vacA+), we have observed a reciprocal increase in the expression of SDF‐1 mRNA. Both, IL‐6 and SDF‐1 mRNA signals have been shown to potentiate each other, thus accelerating the cytokine network in tumor tissues.^64^ Consistently, we have observed an increase in mRNAs for IL‐8 suggesting that both, IL‐8 and SDF‐1 could contribute to phenotypic changes observed in myofibroblasts cocultured with *H. pylori*. SDF‐1 and IL‐8 can synergistically increase endothelial cell proliferation and migration, boosting the angiogenic pathway with IL‐8, predominantly induced in gastric cancer.^35^, ^38^, ^64^ The secretion of IL‐6, FAP, and IL‐8 by CAFs plays pivotal role in macrophage differentiation or M2 polarization resulting in an immunosuppressive microenvironment.^36^

We have previously reported that mRNA for HIF‐1α, the another potent tumor activator,^65^ is upregulated in fibroblasts infected by *H. pylori* (cagA+vacA+) strain.^10^ HIF‐1α is known to induce transcription of more than 60 genes and to play an important role in CAFs activation and release of a great number of proangiogenic factors including SDF‐1, IL‐8, and VEGF^35^, ^65^ also examined in our present study. CAFs produce a variety of ECM proteins such as fibronectin, TNC, and collagens, the structural components that make up connective tissue and contribute to the dense fibrous nature of solid tumors.^66^, ^67^, ^68^, ^69^, ^70^, ^71^ The metabolism of collagen is deregulated in cancer by the increased expression, turnover, elevated deposition, and altered organization with enhanced matrix metalloproteinases (MMPs) activity, all implicated in tumor progression.^67^, ^68^ Furthermore, ECM stiffening was required to corporate with TGFβ to induce EMT in human breast tumor cells, further strengthening the notion that mechanical properties of the tumor microenvironment are key factors regulating EMT and promoting tumor progression.^69^

TNC belongs to an antiadhesive or adhesion‐modulating proteins that have been shown to interact with fibronectin and to promote the migratory activities of various cell types including fibroblasts, epithelial, and cancer cells.^71^ The elevated expression of IL‐6, IL‐8, and SDF‐1 mRNAs could be mediated by the increased deposition of TNC. The *H. pylori* strain (cagA+vacA+) enhanced mRNA expression for TNC, collagen I and III possibly due to activation of STAT3, NFκB, and HIF‐1α pathways. Moreover, the increased expression of HGF and TGFβ both considered as the essential promigrative growth factors secreted by CAFs^35^, ^40^, ^72^ has been observed in our *H. pylori* (cagA+vacA+)‐infected fibroblasts. These results indicate that the *H. pylori*‐derived cytotoxic antigens cagA and vacA may be essential for fibroblast differentiation and myofibroblast formation and might act as prerequisite for progression of normal fibroblasts into CAFs. The tumor invasion and metastasis are initiated by EMT program involving cells acquiring mesenchymal phenotype characterized with decreased cell‐to‐cell adhesion, increased motility and invasive properties. Such cells once detached from primary tumor invade surrounding tissues through collective or individual cell migration.^3^, ^20^, ^21^, ^22^, ^23^, ^24^, ^25^, ^26^, ^67^, ^68^, ^69^, ^70^, ^71^, ^73^ Therefore, we propose that fibroblasts infected with *H. pylori* (cagA+vacA+) likely acquired characteristics of CAFs with changes in morphology and elevated HGF, TGFβ, and additionally IL‐6 and SDF‐1 release, which may enhance EMT program in epithelial gastric cells.

In consequence, the alteration such as reduction in cell‐to‐cell adhesion molecule E‐cadherin, the upregulation of more “plastic” mesenchymal proteins such as vimentin, N‐cadherin, and α‐SMA were facilitated. The E‐cadherin to N‐cadherin “switch” exerts critical role in cancer progression being essential for enhanced cell motility and migration.^26^ Our results are in keeping with these observations as epithelial RGM‐1 cells cocultured with differentiated fibroblasts revealed the fall in expression of E‐cadherin mRNA and the rise in N‐cadherin and vimentin mRNA expression. Downregulation of E‐cadherin and initiation of EMT can be triggered by key EMT‐inducing transcription factors including Snail and Twist. Since Snail directly represses epithelial markers including E‐cadherin, it can also upregulate markers of the mesenchymal phenotype and facilitate EMT processes during tumor progression.^23^, ^74^ The Snail expression associated with features of EMT has been observed, for example, in colon and esophageal cancers.^75^

Twist has been shown to act as another potent E‐cadherin repressor and N‐cadherin activator.^23^, ^24^, ^74^ Both, Snail and Twist are downstream in signaling pathways activated by TGFβ, integrins, IL‐6, FGF, and HGF.^3^, ^23^, ^76^ In agreement with these findings, we provide evidence that *H. pylori*‐induced differentiation and activation of fibroblasts with rise in HGF and TGFβ mRNA expression were accompanied by increase in HGFR, TGFβR, FGFR, Snail, and Twist mRNA expression in RGM‐1 cells. The mechanism of activation of cell proliferation and survival pathways through integrin interactions with downstream molecules is considered as crucial for cell motile function and survival.^77^, ^78^ That is why we have decided to analyze the expression of β1‐integrin mRNA, which as shown in our present work, has also increased. During cancer differentiation and metastasis processes, upregulation of integrins has been linked to cancer invasiveness.^79^, ^80^ Among others, the β1‐integrin subunit is expressed in metastatic cells and can be considered as indicator for metastasis.^81^ Xu et al^82^ observed that TGF‐β1 had obviously increased the expression of integrin α5β1. They assumed that TGF‐β1‐promoted EMT and cell adhesion contribute to the TGF‐β1 enhanced cell migration in SMMC‐7721 cells.^82^ All these observations support our notion that the epithelial RGM‐1 cells underwent the EMT process.

Changes in mRNA expression of various mediators observed in RGM‐1 cells in our present study are strengthened by an evidence from contrast‐phase microscopy showing changes in cell morphology characterized by cell elongation with prominent protrusions and distorted continuity of monolayers. Taken together, our present study underlines the importance of *H. pylori* (cagA+vacA+) affecting fibroblasts differentiation in the direction of cells bearing CAFs characteristics, likely to initiate EMT process in epithelial RGM‐1 cells as reflected by their activation and phenotypical changes. Our next goal will be to confirm the observed changes on EMT induced by *H. pylori* at the level of protein expression.

## DISCLOSURES OF INTERESTS

There is no actual or potential conflict of interest including financial, personal, or other relationship with other people or organizations associated with article.

## APPENDIX I

**Table A1:** Rat oligonucleotide primers for detection of mRNA by RT‐PCR, annealing temperature, and size of PCR products employed in the experimental protocol

Gene	Primer sequence	Annealing temperature	Size of PCR product
α‐SMA	Forward 5′‐CAT CAG GCA GTT CGT AGC TC‐3′	60°C	525 bp
	Reverse: 5′‐CGA TAG AAC ACG GCA TCA TC‐3′		
Collagen I	Forward 5′‐GGC AAC AGT CGA TTC ACC‐3′	60°C	177 bp
	Reverse 5′‐AGG GCC AAT GTC CAT TCC G‐3′		
Collagen III	Forward 5′‐TGC AGG GCC TGG ACT ACC‐3′	60°C	498 bp
	Reverse 5′‐CCT GGA CCT CAG GGT ATC‐3′		
COX‐2	Forward 5′‐ACA ACA TTC CCT TCC TTC‐3′	56°C	201 bp
	Reverse 5′‐CCT TAT TTC CTT TCA CAC C‐3′		
Bcl‐2	Forward 5′‐CTG CCA ACC CAC CCT GGT CT‐3′	55°C	235 bp
	Reverse 5′‐TGG CAG CTG ACA TGT TTT CT‐3′		
18s‐RNA	Forward 5′‐GTT GGT TTT GAT CTG ATA AAT GC‐3′	60°C	143 bp
	Reverse 5′‐CAT TAA ATC AGT TAT GGT TCC TT TG‐3′		
E‐cadherin	Forward 5′‐AAC GAG GGC ATT CTG AAA ACA‐3′	60°C	75 bp
	Reverse 5′‐CAC TGT CAC GTG CAG AAT GTA CT‐3′		
HGFR	Forward 5′‐TCC AGC TGT TGC AGG GAA G‐3′	60°C	66 bp
	Reverse 5′‐GGC GTG CCA ACA TCG C‐3′		
FGFR	Forward 5′‐CTG GGC AGC AAC GTG GA‐3′	60°C	66 bp
	Reverse 5′‐CAG CCA CTG GAT GTG AGG C‐3′		
Twist	Forward 5′‐GCC GGA GAC CTA GAT GTC ATT‐3′	60°C	185 bp
	Reverse 5′‐GGC CTG TCT CGC TTT CTC TT‐3′		
Snail	Forward 5′‐CTG GGC GCT CTG AAG ATG CA‐3′	60°C	250 bp
	Reverse 5′‐GGA GCA GCC AGA CTC TTG GTG T‐3′		
N‐cadherin	Forward 5′‐GAC CCA GAA GAT GAT GTA AG‐3′	60°C	171 bp
	Reverse 5′‐CTC AGC GTG GAT AGG C‐3′		
FSP 1	Forward 5′‐ACC TCT CTG TTC AGC ACT TCC‐3′	60°C	131 bp
	Reverse 5′‐GAA CTT GTC ACC CTC GTT GC‐3′		
Vimentin	Forward 5′‐GCA AGG ATT CCA CTT TAC GTT CA AGG‐3′	62°C	324 bp
	Reverse 5′‐GGT GGA TCA GCT CAC CAA TGA CA AG‐3′		
Integrin β1	Forward 5′‐GAG AGA GAT TAC TTC AGA C‐3′	60°C	303 bp
	Reverse 5′‐AGC AGT CGT GTT ACA TTC‐3′		
FAP	Forward 5′‐AGC CAT ATG GGG ATG GTC CT‐3′	60°C	154 bp
	Reverse 5′‐TGT TGG GAG GCC CAT GAA TC‐3′		
VEGF	Forward 5′‐TGC ACC CAC GAC AGA AGG GG A‐3′	60°C	436 bp
	Reverse 5′‐TCA CCG CCT TGG CTT GTC AC A‐3′		364 bp
TGFβR	Forward 5′‐TGT GGC AGA GCG CTT CAG T‐3′	60°C	95 bp
	Reverse 5′‐TGT TCA GGG AGC CGT CTT CT‐3′		
SDF‐1	Forward 5′‐AAA CCA GTC AGC CTG AGC TAC‐3′	60°C	129 bp
	Reverse 5′‐TTA CTT GTT TAA AGC TTT CTC‐3′		
HGF	Forward 5′‐TCT TGG TGT CAT TGT TCC TG‐3′	60°C	157 bp
	Reverse 5′‐CCA TGG ATG CTT CAA ATA CA‐3′		
IL‐6	Forward 5′‐GAC TGA TGT TGT TGA CAG CCA CTG C‐3′	60°C	509 bp
	Reverse 5′‐TAG CCA CTC CTT CTG TGA CTC TAA CT‐3′		
TGF β1	Forward 5′‐ACT GAA GCG AAA GCC CTG TA‐3′	58°C	501 bp
	Reverse 5′‐CTG TCC AAA CTA AGG CTC GC‐3′		
Ten C	Forward 5′‐AGG CCA CTG AGT ACG AAA TT‐3′	55°C	360 bp
	Reverse 5′‐GAC CAT CGA GAG GCT GTG ATT‐3′		
Ki‐67	Forward 5′‐AAC CAG GAC TTT GTG CTC TGT AA‐3′	60°C	209 bp
	Reverse 5′‐CTC TTT TGG CTT CCA TTT CTTC‐3′		
Bax	Forward 5′‐CGT CCA ACC CAC CCT GGT CT‐3′	55°C	195* *bp
	Reverse 5′‐TGG CAG CTG ACA TGT TTT CTG AC‐3′		
IL‐8	Forward 5′‐CCC CCA TGG TTC AGA AGA TTG‐3′	60°C	113 bp
	Reverse 5′‐TTGTCAGAAGCCAGCGTTCAC‐3′		

**Table A2:** The sequence, annealing temperature, and size of PCR products of oligonucleotide primers for detection of rat and specific *Helicobacter pylori* mRNAs by RT‐PCR for selection of *H. pylori* (cagA−vacA−) and its expression relevance in fibroblasts

Gene	Primer sequence	Annealing temperature	Size of PCR product
cagA	Forward 5′‐ATA ATG CTA AAT TAG ACA ACT TGA GCG‐3′	60°C	298 bp
	Reverse 5′‐TTA GAA TAA TCA ACA AAC ATC ACG CCA‐3′		
vacA m1	Forward 5′‐GGT CAA AAT GCG GTC ATG G‐3′	57°C	290 bp
	Reverse 5′‐CCA TTG GTA CCT GTA GAA AC‐3′		
vacA m2F	Forward 5′ GGA GCC CCA GGA AAC ATT G‐3′	57°C	352 bp
	Reverse 5′‐CAT AAC TAG CGC CTT GCA C‐3′		
vacA s1/s2	Forward 5′‐ATG GAA ATA CAA CAA ACA CAC‐3′	57°C	259/286 bp
	Reverse 5′‐CTG CTT GAA TGC GCC AAA C‐3′		
HSP70	Forward 5′‐CAA GAA TGC GCT CGA GTC CTA‐3′	60°C	124 bp
	Reverse 5′‐GGA GAT GAC CTC CTG GCA CTT‐3′		
β‐Actin	Forward 5′‐TTG TAA CCA ACT GGG ACG ATA TGG‐3′	54°C	764 bp
	Reverse 5′‐GAT CTT GAT CTT CAT GGT GCT AGG‐3′		
HIF‐1α	Forward 5′‐TCT GGA CTC TCG CCT CTG‐3′	61°C	510 bp
	Reverse 5′‐GCT GCC CTT CTG ACT CTG‐3′		
